# Supplementary material for: The Soybean High Density ‘Forrest’ by ‘Williams 82’ SNP-Based Genetic Linkage Map Identifies QTL and Candidate Genes for Seed Isoflavone Content
Source: Plants (Basel). 2021 Sep 27;10(10):2029. doi: 10.3390/plants10102029 (PMC8541105; doi:10.3390/plants10102029)
Supplement: Supplementary file 1 [file plants-10-02029-s001.zip › plants-1372056-supplementary.pdf]

Supplementary Tables and Figures.

(A) QTL Identified in Spring Lake, NC (2018)

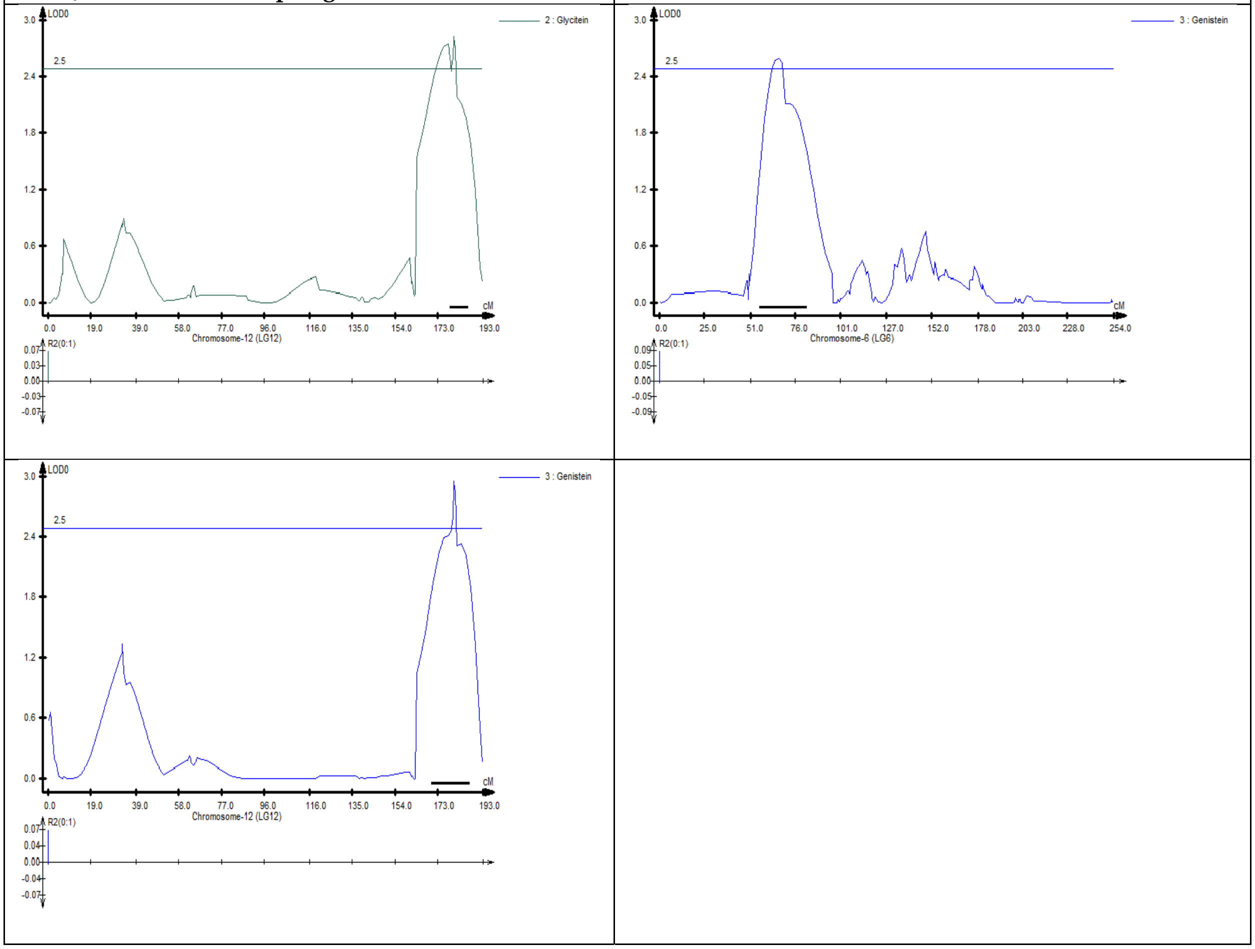

(B) QTL Identified in Carbondale, IL (2020)

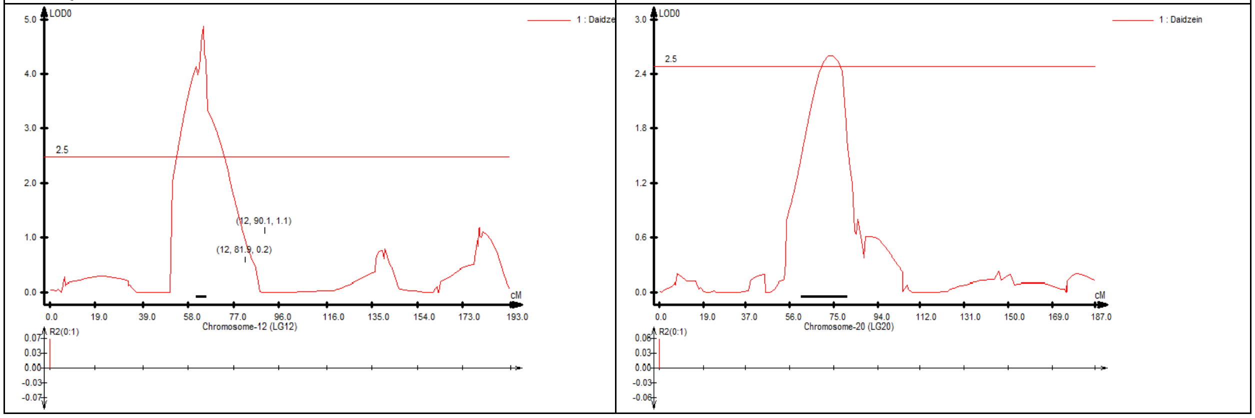

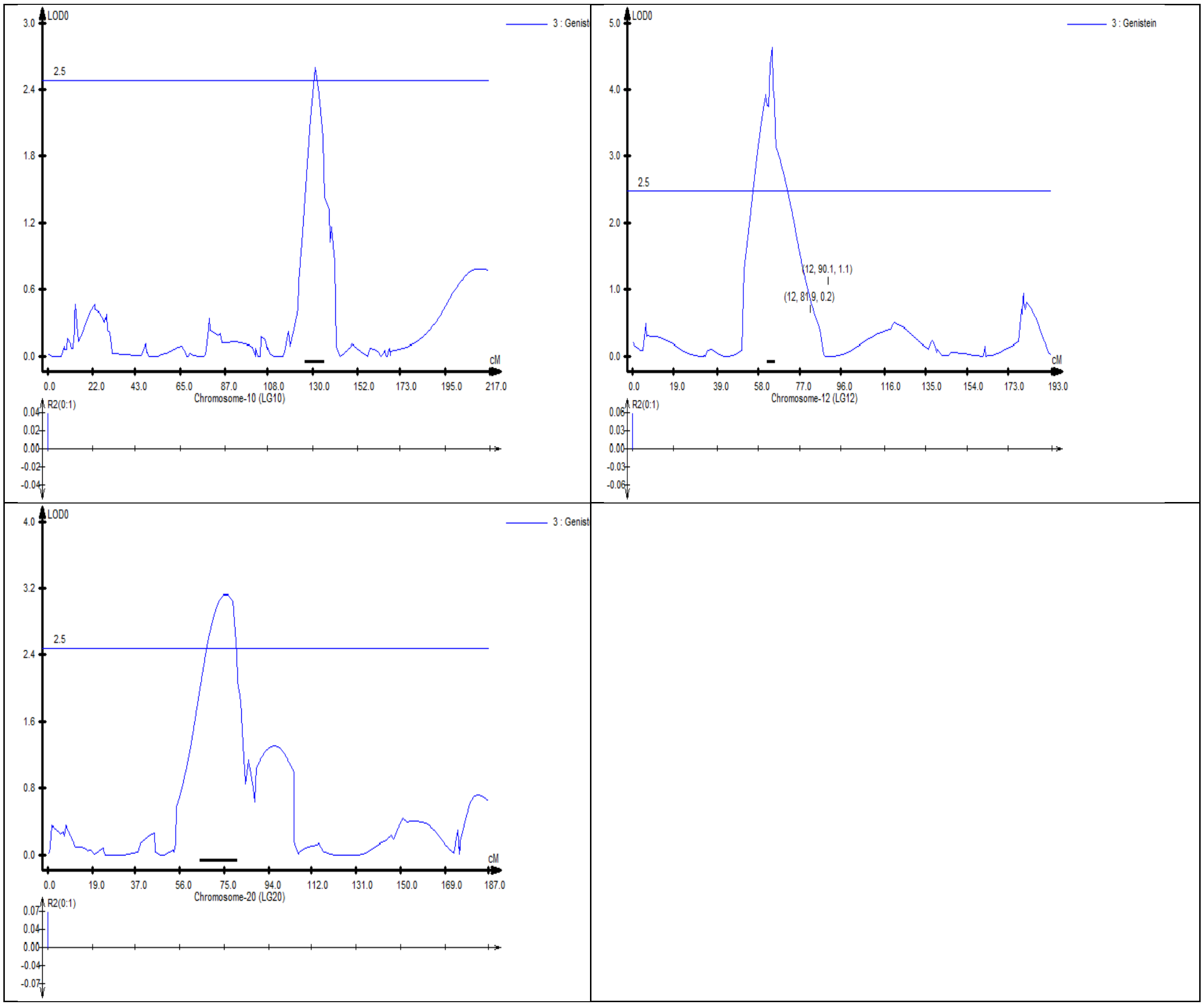

Figure S1. Positions of major QTL (LOD > 2.5) that control seed genistein (qGEN), daidzein (qDAID), and glycitein (qGLY) contents identified in the FXW82 RIL population. (A) QTL identified in Spring Lake, NC (2018) and (B) QTL identified in Carbondale, IL (2020).

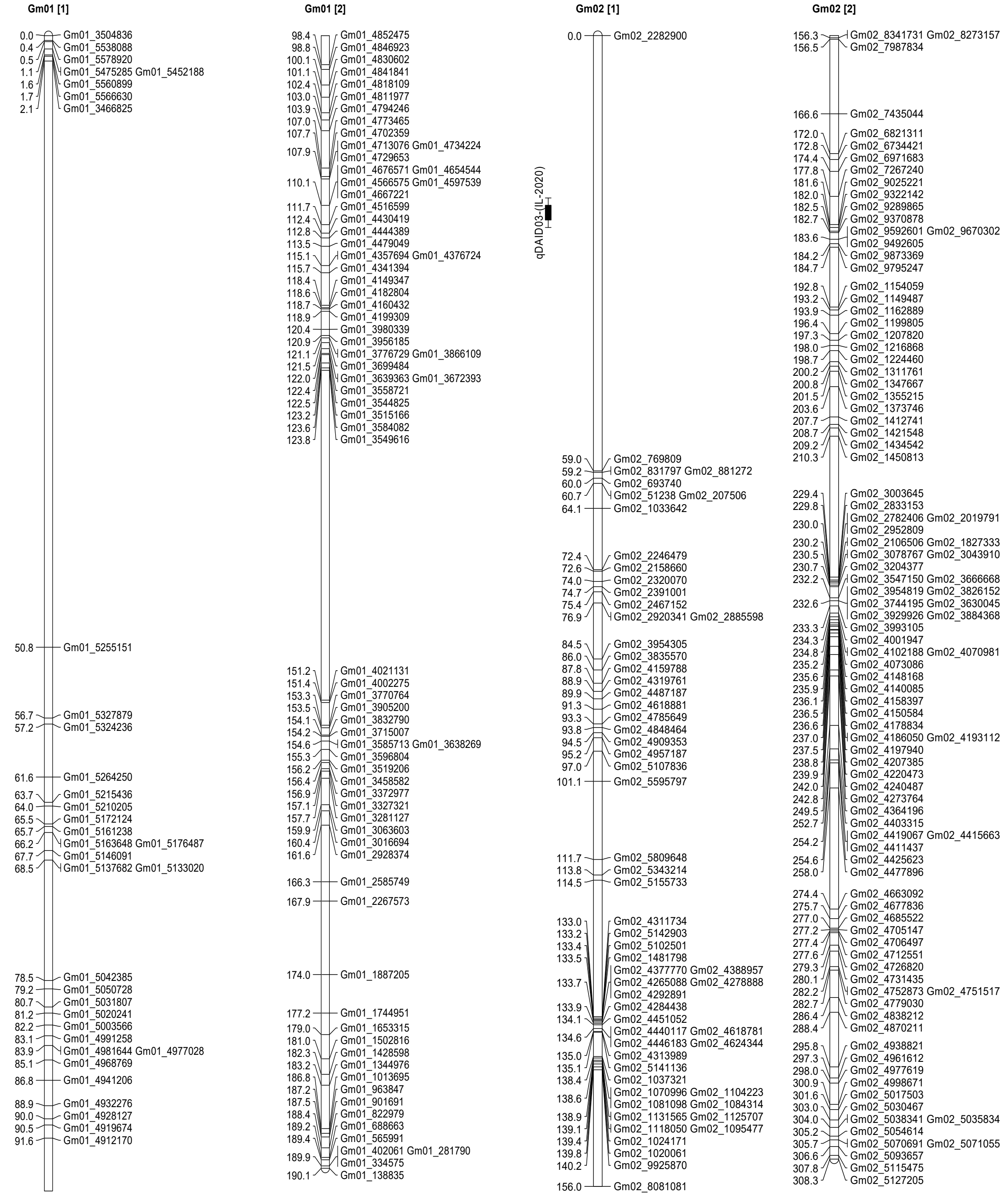

Figure S2. The SNP-Based genetic linkage map of Forrest by Williams 82 recombinant inbred line (RIL) population (n=306) of soybean. Chr.s were drawn using MapChart 2.2 (Voorrips, 2002 [41]). Positions of QTL that control seed genistein (qGEN), daidzein (qDAID), and glycitein (qGLY) contents are indicated with black bars on Chrs. 2, 4, 5, 6, 10, 12, 15, 19, and 20. QTL names are followed by a number, location, and year in which they are identified. For example, qGEN01-(NC-2018).

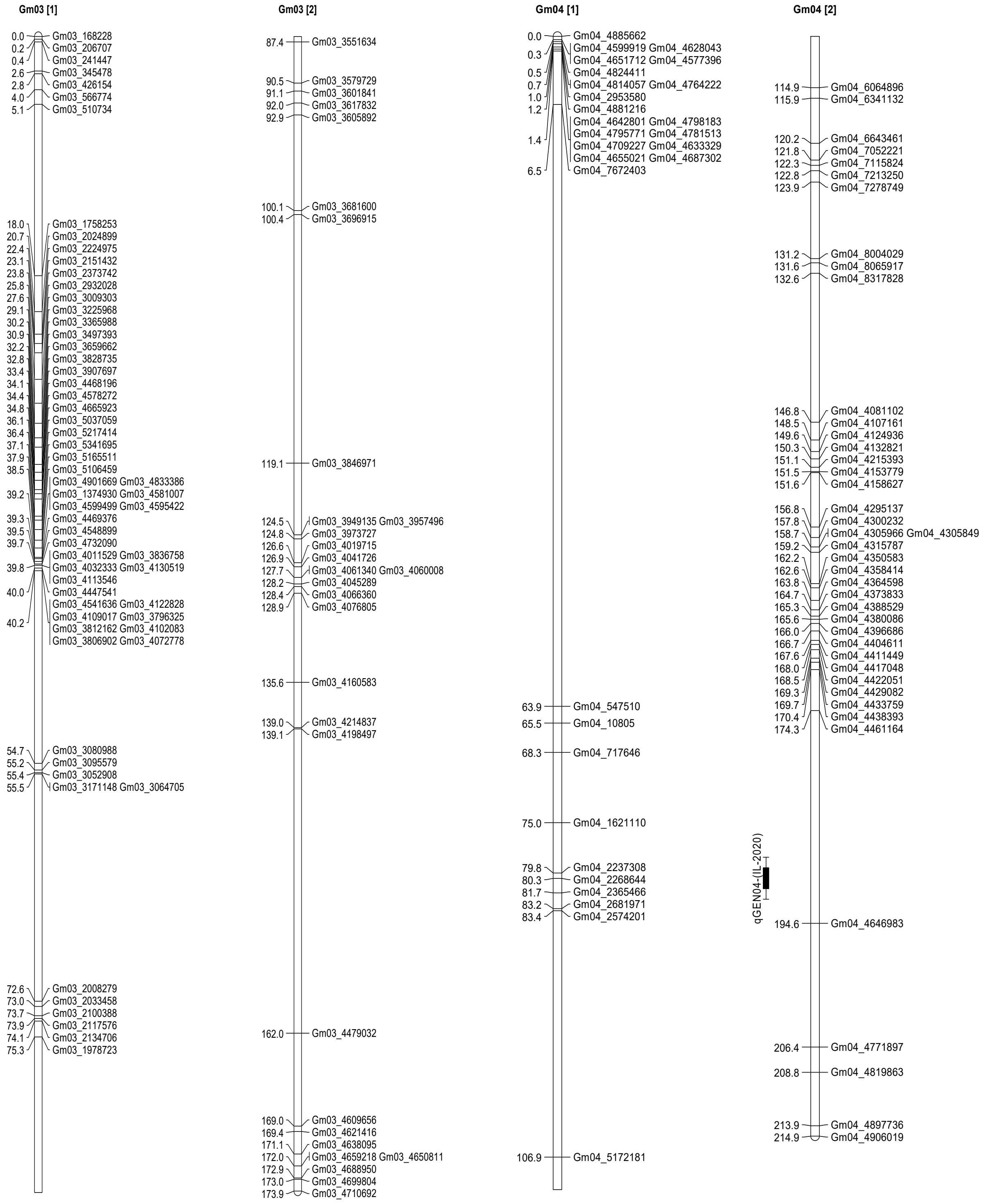

Figure S2. Continued.

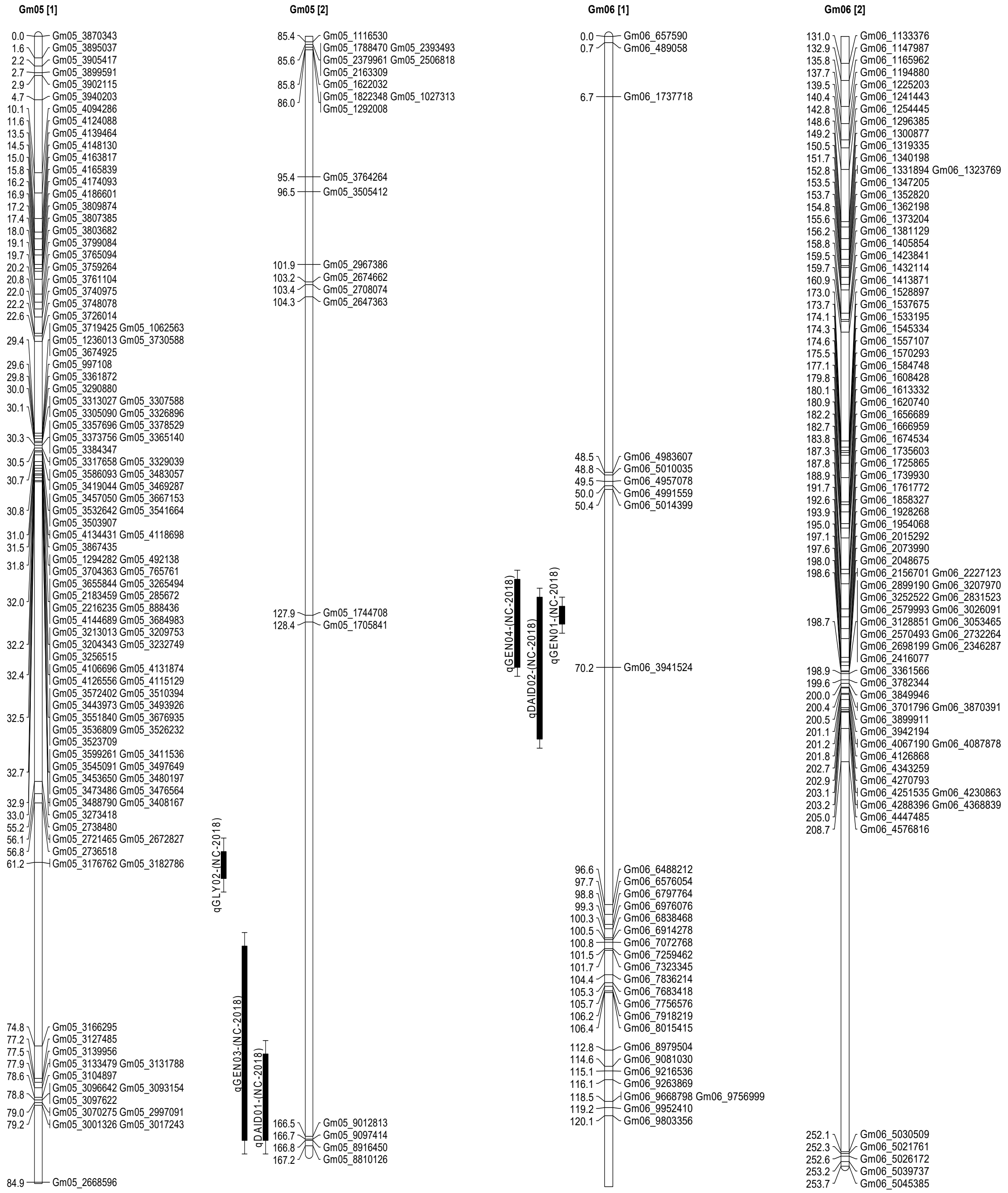

Figure S2. Continued.

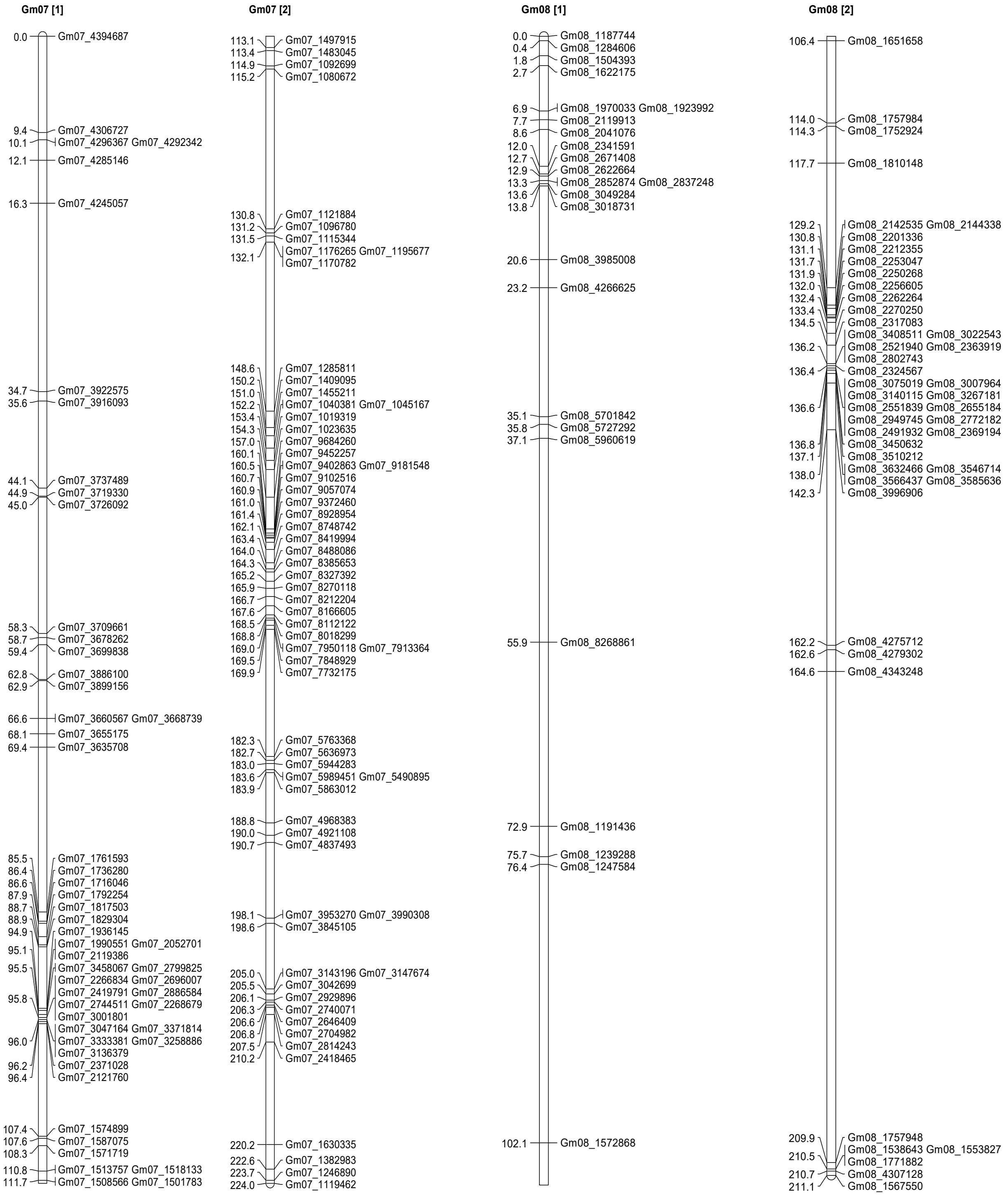

Figure S2. Continued.

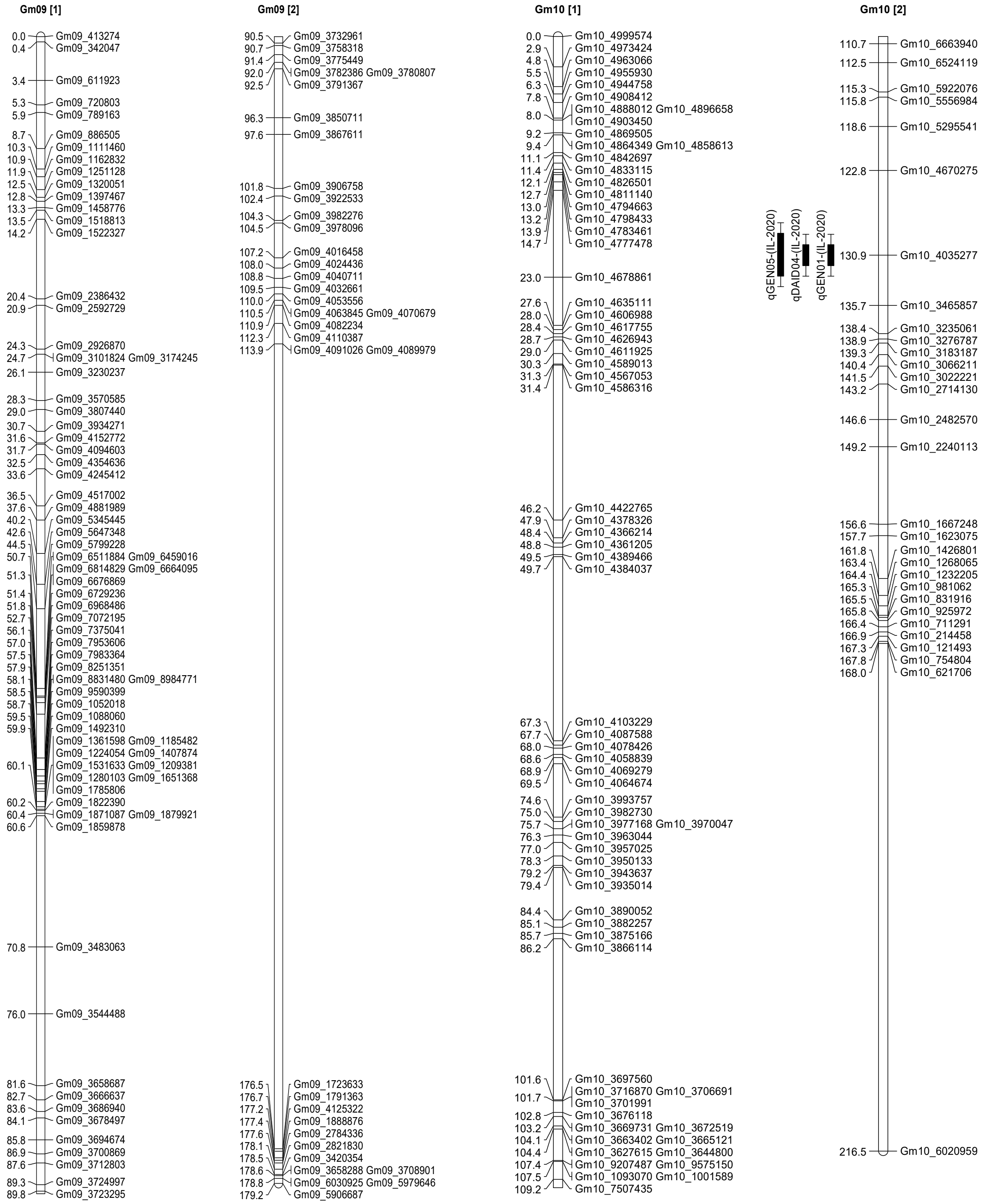

Figure S2. Continued.

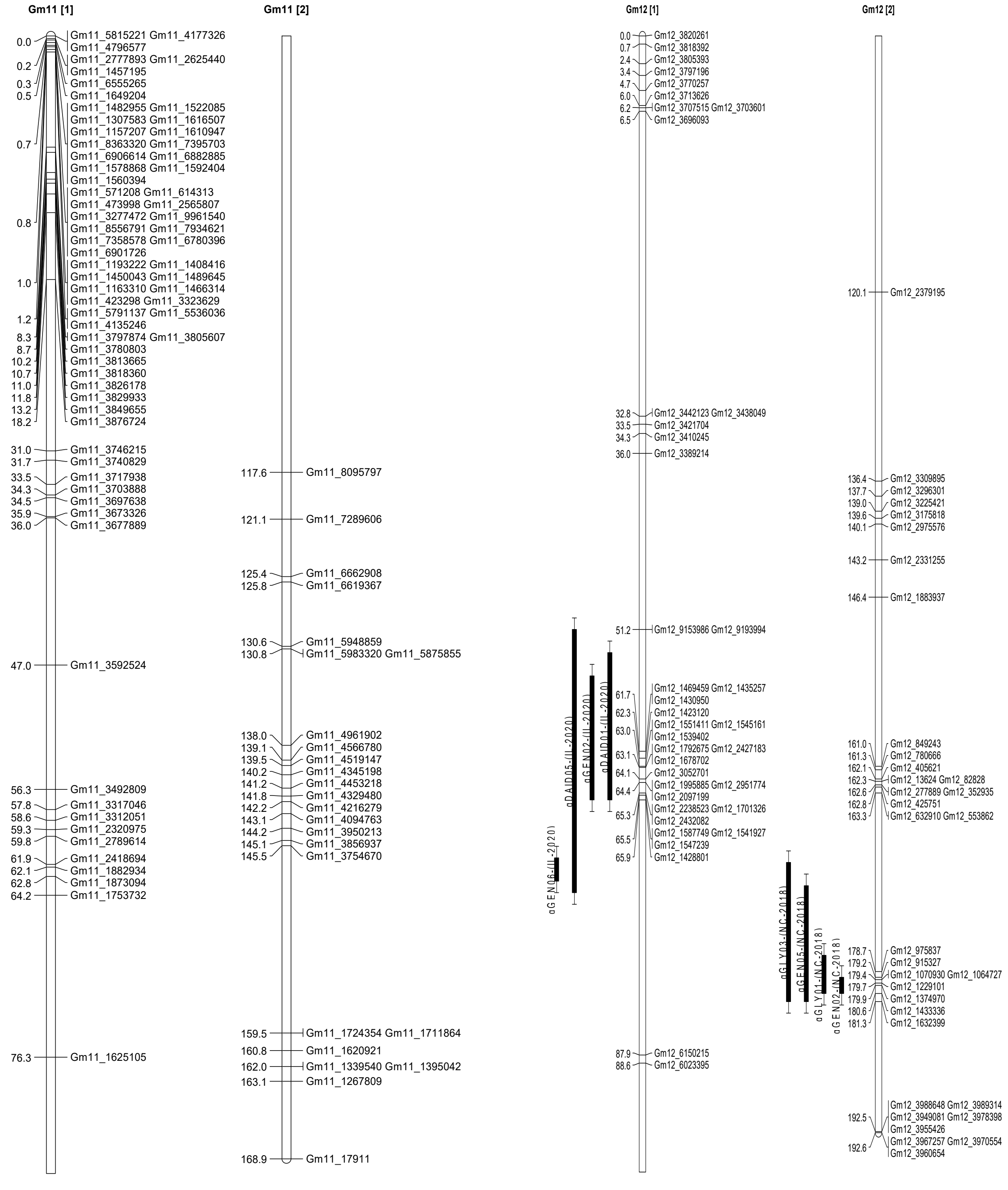

Figure S2. Continued.

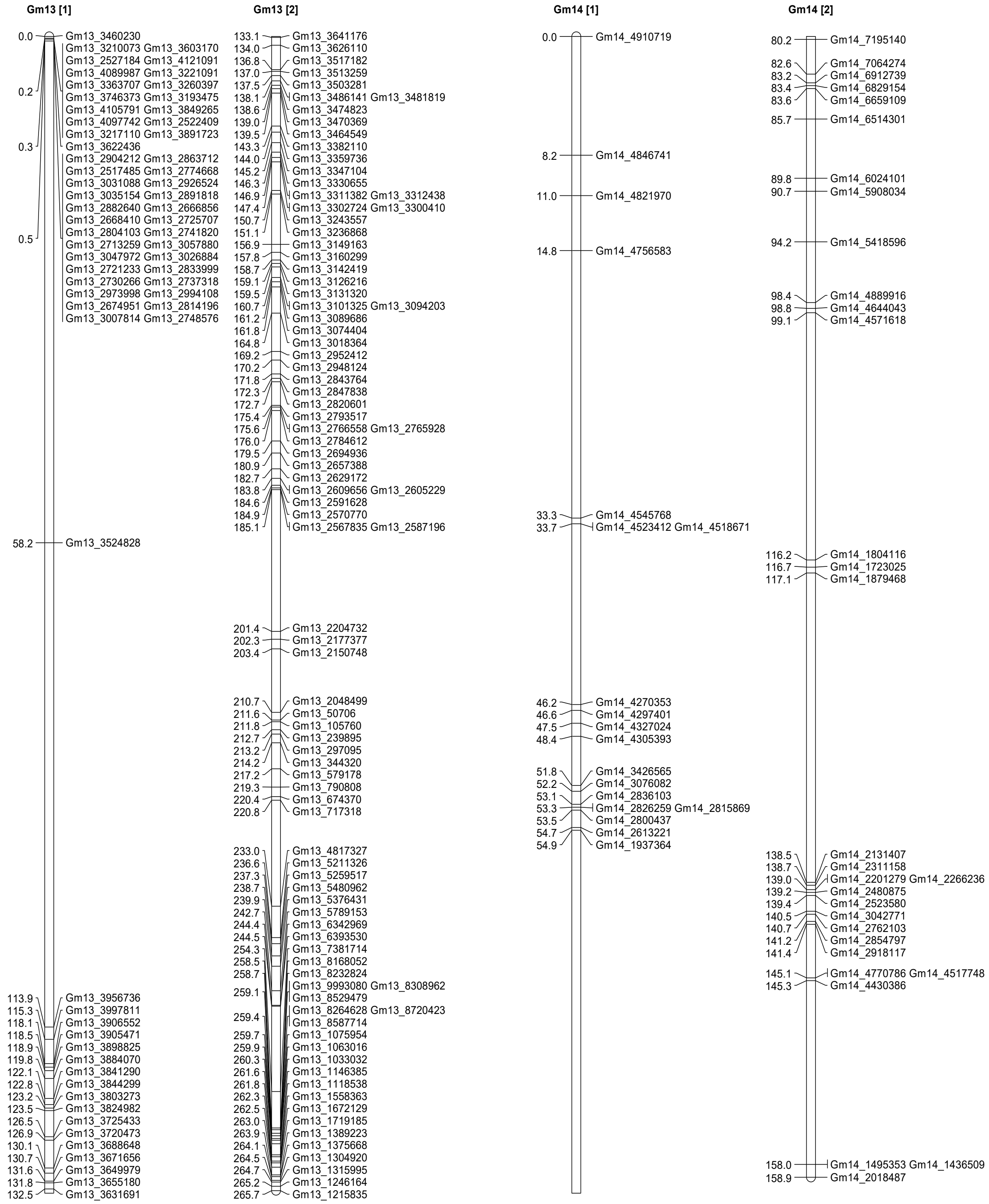

Figure S2. Continued.

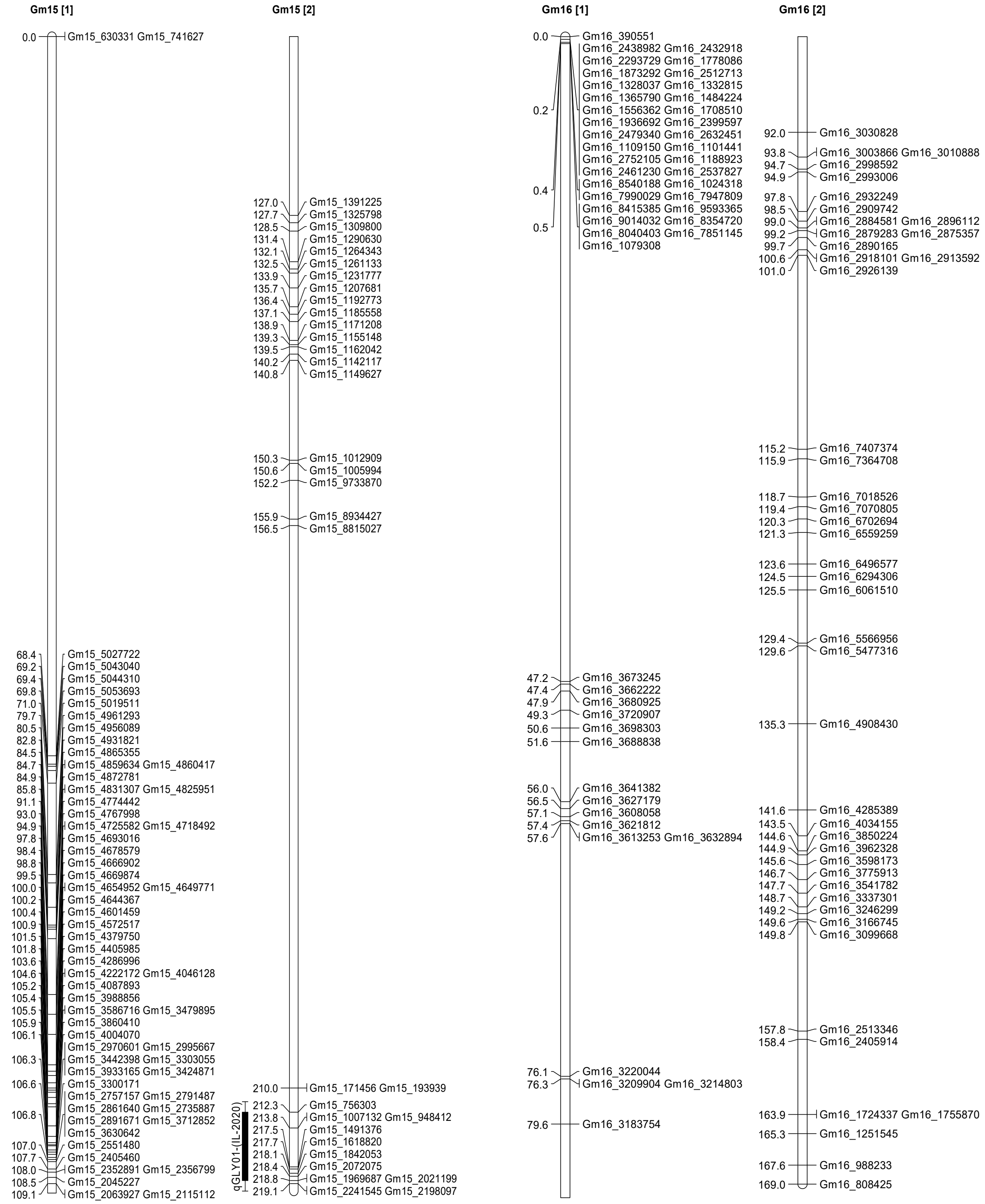

Figure S2. Continued.

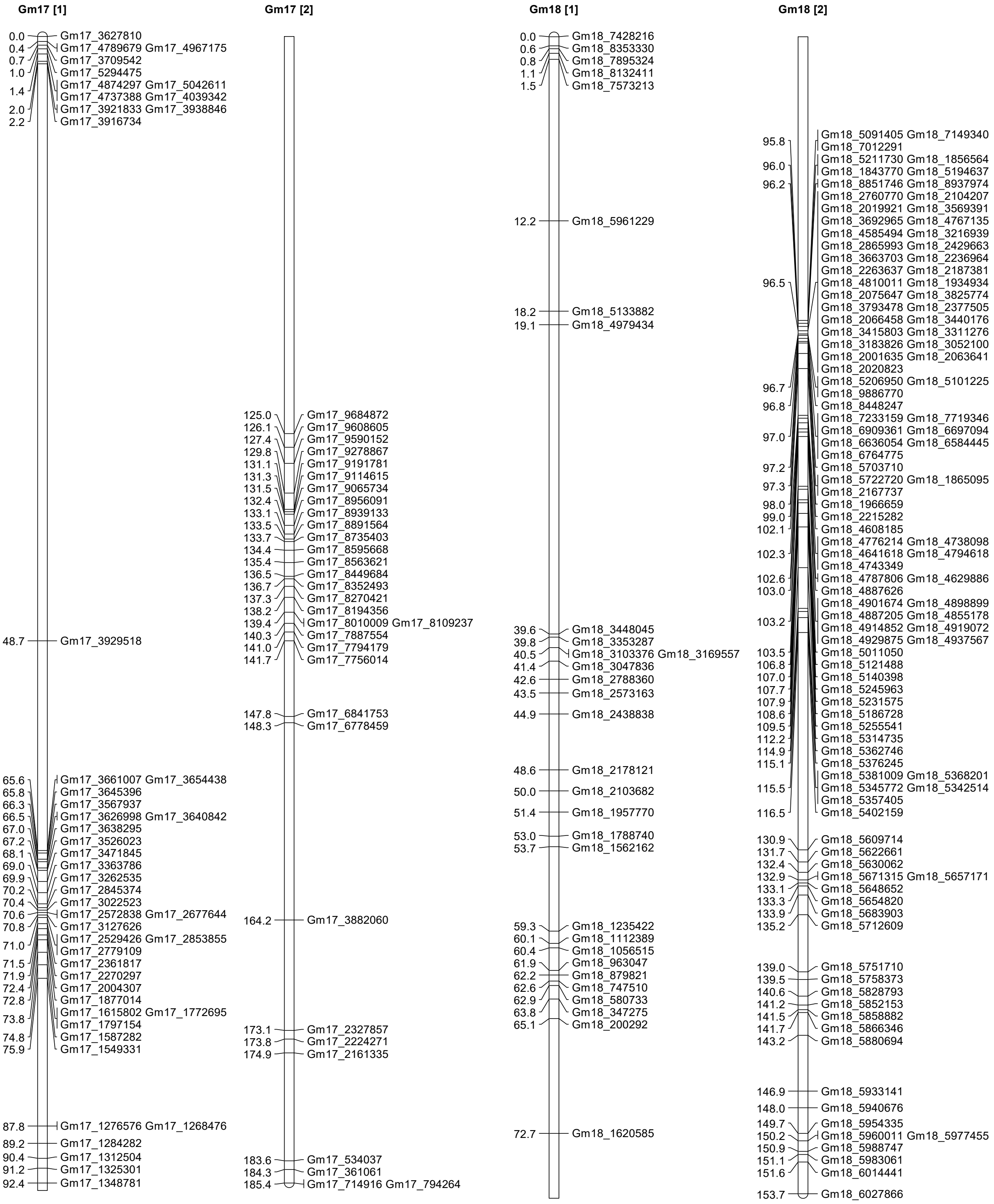

Figure S2. Continued.

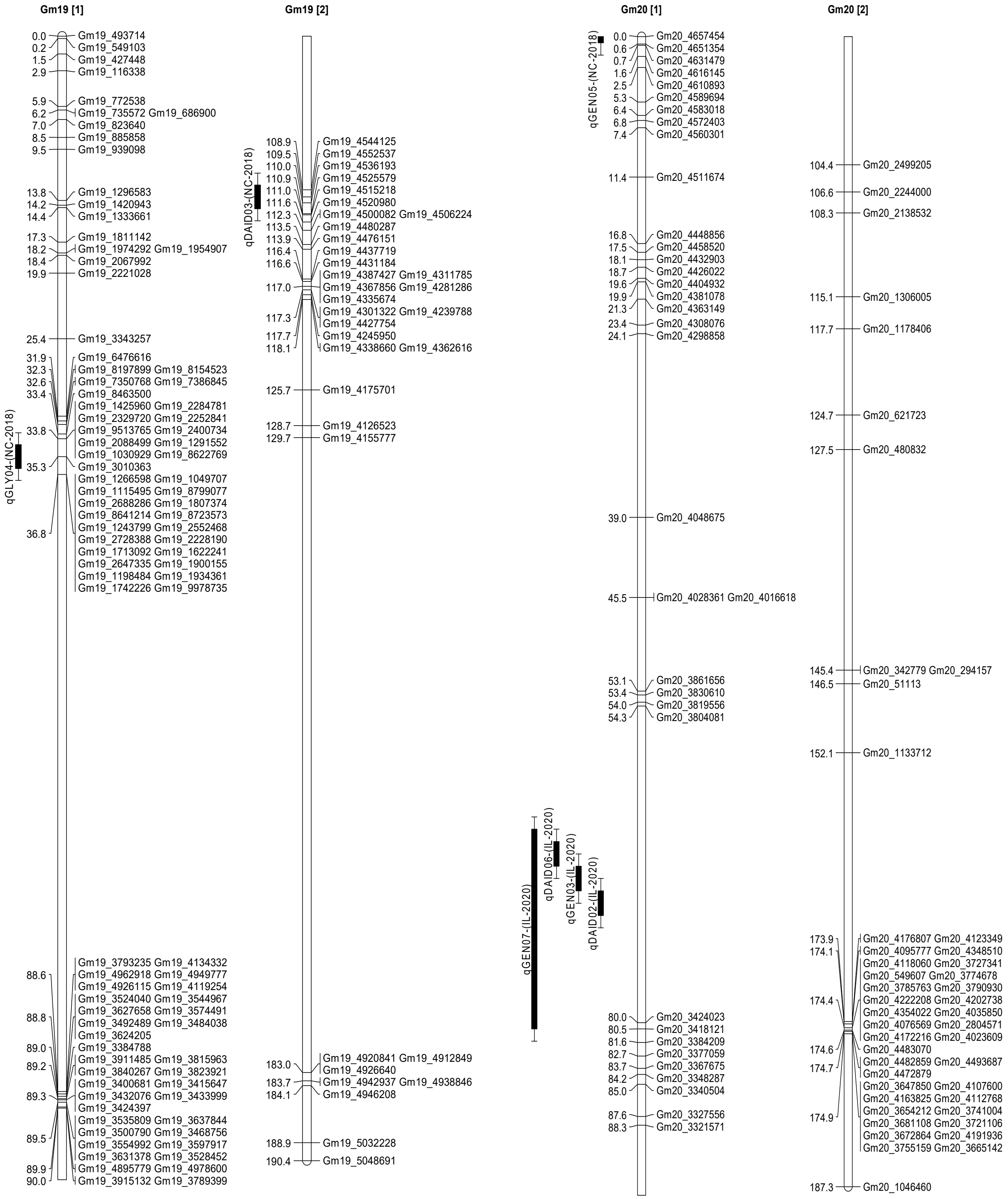

Figure S2. Continued.

Table S1. Candidate genes involved in soybean isoflavone biosynthetic pathways and their Phytozome annotation. These 130 candidate genes are identified in all 20 chrs.

Soybean Gene	Phytozome Annotation	Chr	Start	End
Glyma.01G239300	2-hydroxyisoflavanone dehydratase	Chr01	56350646	56352389
Glyma.01G239500	2-hydroxyisoflavanone dehydratase	Chr01	56360162	56361708
Glyma.01G239400	2-hydroxyisoflavanone dehydratase	Chr01	56356064	56357252
Glyma.20G143400	2-hydroxyisoflavanone dehydratase	Chr20	38199806	38201866
Glyma.10G250200	2-hydroxyisoflavanone dehydratase	Chr10	47837207	47839071
Glyma.01G239600	2-hydroxyisoflavanone dehydratase (HID)	Chr01	56367073	56368854
Glyma.13G173400	2-hydroxyisoflavanone synthase / Isoflavonoid synthase	Chr13	28738547	28739205
Glyma.11G070500	2'-hydroxyisoflavone reductase	Chr11	5273807	5275911
Glyma.01G172900	2'-hydroxyisoflavone reductase	Chr01	51020276	51025649
Glyma.01G172600	2'-hydroxyisoflavone reductase	Chr01	50980302	50982573
Glyma.10G292200	3,9-dihydroxypterocarpan 6A-monooxygenase / 3,9-dihydroxypterocarpan 6A-hydroxylase	Chr10	51006927	51009538
Glyma.19G144700	3,9-dihydroxypterocarpan 6A-monooxygenase / 3,9-dihydroxypterocarpan 6A-hydroxylase	Chr19	40579736	40582424
Glyma.02G067900	4'-methoxyisoflavone 2'-hydroxylase / Isoflavone 2'-monooxygenase	Chr02	5986285	5987684
Glyma.08G089500	4'-methoxyisoflavone 2'-hydroxylase / Isoflavone 2'-monooxygenase	Chr08	6765669	6768017
Glyma.20G241500	chalcone isomerase (E5.5.1.6)	Chr20	47259311	47261877
Glyma.13G262500	Chalcone isomerase / Chalcone--flavanone isomerase	Chr13	36603843	36608523
Glyma.20G241600	chalcone isomerase	Chr20	47264269	47266795
Glyma.20G241700	chalcone isomerase	Chr20	47267106	47268550
Glyma.19G105100	chalcone synthase (CHS)	Chr19	35466392	35469297
Glyma.09G075200	chalcone synthase (CHS)	Chr09	8145494	8147595
Glyma.11G011500	chalcone synthase (CHS)	Chr11	802453	804663
Glyma.02G130400	chalcone synthase (CHS)	Chr02	13399253	13401493
Glyma.01G091400	chalcone synthase (CHS)	Chr01	27621455	27623628
Glyma.08G109400	chalcone synthase (CHS)	Chr08	8391364	8394840
Glyma.08G109300	chalcone synthase (CHS)	Chr08	8387509	8391327
Glyma.08G109500	chalcone synthase (CHS)	Chr08	8397944	8399751
Glyma.08G110500	chalcone synthase (CHS)	Chr08	8504479	8506020
Glyma.01G228700	chalcone synthase (CHS)	Chr01	55659010	55660950
Glyma.05G153200	chalcone synthase (CHS)	Chr05	34687009	34693243
Glyma.06G118500	chalcone synthase (CHS)	Chr06	9644661	9650144
Glyma.08G109200	chalcone synthase (CHS)	Chr08	8384742	8386542
Glyma.08G110300	chalcone synthase (CHS)	Chr08	8475793	8477410
Glyma.08G110700	chalcone synthase (CHS)	Chr08	8513952	8515719
Glyma.08G110900	chalcone synthase (CHS)	Chr08	8517799	8519303
Glyma.11G077200	Chalcone-flavanone isomerase family protein	Chr11	5785880	5787797
Glyma.01G166300	Chalcone-flavanone isomerase family protein	Chr01	50402188	50404181
Glyma.04G222400	Chalcone-flavanone isomerase family 3-related	Chr04	49304081	49306117
Glyma.06G143000	Chalcone-flavanone isomerase family 3-related	Chr06	11642031	11644022
Glyma.12G067000	Cytochrome P450 CYP2 subfamily	Chr12	4909073	4911905
Glyma.12G067100	Cytochrome P450 CYP2 subfamily	Chr12	4919960	4922998
Glyma.19G151100	Dirigent protein 1-related	Chr19	41141615	41142515

Glyma.13G173500	Dirigent protein 1-related	Chr13	28761503	28763755
Glyma.10G250300	Dirigent protein 1-related	Chr10	47841780	47843423
Glyma.19G151200	Dirigent protein 1-related	Chr19	41150640	41151405
Glyma.03G147700	Dirigent protein 1-related	Chr03	36329060	36329804
Glyma.08G103900	Flavonoid 3',5'-methyltransferase	Chr08	7972204	7974378
Glyma.18G267800	Flavonoid 4'-O-methyltransferase / Isoflavone O-methyltransferase	Chr18	55192276	55194120
Glyma.08G246700	Flavonoid 4'-O-methyltransferase / Isoflavone O-methyltransferase	Chr08	21363198	21364914
Glyma.10G176300	Flavonoid 4'-O-methyltransferase / Isoflavone O-methyltransferase	Chr10	41003284	41004506
Glyma.04G227700	Flavonol 3-O-methyltransferase	Chr04	49704598	49706834
Glyma.06G137300	Flavonol 3-O-methyltransferase	Chr06	11237072	11239469
Glyma.06G137100	Flavonol 3-O-methyltransferase	Chr06	11225188	11228664
Glyma.06G110600	Flavonol synthase (FLS)	Chr06	8941619	8944385
Glyma.13G082300	Flavonol synthase (FLS)	Chr13	19154278	19159357
Glyma.06G128200	Flavonol synthase / FLS	Chr06	10543911	10545747
Glyma.07G150900	Flavonol synthase / FLS	Chr07	18177981	18182051
Glyma.07G180700	Flavonol synthase / FLS	Chr07	34888861	34890596
Glyma.18G201900	Flavonol synthase / FLS	Chr18	48192545	48196624
Glyma.07G179800	Flavonol synthase / FLS // Flavanone 3-dioxygenase / Naringenin,2-oxoglutarate:oxygen oxidoreductase (3-hydroxylating)	Chr07	34505933	34509654
Glyma.14G163300	Flavonol synthase 3-related	Chr14	40082673	40096088
Glyma.20G245100	Homogentisate phytyltransferase / homogentisate geranylgeranyltransferase (HPT, HGGT, ubiA)	Chr20	47561270	47568860
Glyma.14G098100	Homogentisate phytyltransferase / homogentisate geranylgeranyltransferase (HPT, HGGT, ubiA)	Chr14	9259148	9266365
Glyma.17G226600	Homogentisate phytyltransferase / homogentisate geranylgeranyltransferase (HPT, HGGT, ubiA)	Chr17	38105929	38111302
Glyma.10G295300	Homogentisate phytyltransferase / homogentisate geranylgeranyltransferase (HPT, HGGT, ubiA)	Chr10	51250135	51256351
Glyma.16G149300	Isoflavone 2'-hydroxylase	Chr16	30993255	30996642
Glyma.09G049600	Isoflavone 2'-hydroxylase / Isoflavone 2'-monooxygenase	Chr09	4305877	4306472
Glyma.01G190400	Isoflavone 2'-hydroxylase / Isoflavone 2'-monooxygenase	Chr01	52515056	52516522
Glyma.08G089400	Isoflavone 2'-hydroxylase / Isoflavone 2'-monooxygenase	Chr08	6759742	6762340
Glyma.15G156100	Isoflavone 2'-hydroxylase / Isoflavone 2'-monooxygenase	Chr15	13076997	13079333
Glyma.16G149400	Isoflavone 3'-hydroxylase	Chr16	31002000	31004110
Glyma.15G156300	Isoflavone 3'-hydroxylase	Chr15	13098492	13100036
Glyma.19G030500	isoflavone 7-O-glucoside-6''-O-malonyltransferase	Chr19	3779017	3781453
Glyma.13G056100	isoflavone 7-O-glucoside-6''-O-malonyltransferase	Chr13	15382701	15385317
Glyma.09G127200	Isoflavone 7-O-glucosyltransferase	Chr09	31402908	31404677
Glyma.09G127300	Isoflavone 7-O-glucosyltransferase	Chr09	31442521	31444358
Glyma.09G127700	Isoflavone 7-O-glucosyltransferase	Chr09	31720506	31722186
Glyma.16G175600	isoflavone 7-O-glucosyltransferase	Chr16	33709232	33710656
Glyma.16G175200	Isoflavone 7-O-glucosyltransferase	Chr16	33654666	33656275
Glyma.16G175900	isoflavone 7-O-glucosyltransferase	Chr16	33725875	33727942

Glyma.16G175300	Isoflavone 7-O-glucosyltransferase	Chr16	33667521	33669150
Glyma.16G175400	Isoflavone 7-O-glucosyltransferase	Chr16	33682024	33683784
Glyma.16G175500	Isoflavone 7-O-glucosyltransferase	Chr16	33700402	33702163
Glyma.09G094400	Isoflavone 7-O-methyltransferase	Chr09	13197472	13199914
Glyma.08G246900	Isoflavone 7-O-methyltransferase	Chr08	21372889	21373907
Glyma.20G213500	Isoflavone 7-O-methyltransferase	Chr20	44996231	45001230
Glyma.09G094600	Isoflavone 7-O-methyltransferase	Chr09	13248456	13250007
Glyma.18G267500	Isoflavone 7-O-methyltransferase	Chr18	55179516	55181798
Glyma.08G247000	Isoflavone 7-O-methyltransferase	Chr08	21393885	21394984
Glyma.20G213800	Isoflavone 7-O-methyltransferase	Chr20	45012725	45013657
Glyma.10G176500	Isoflavone 7-O-methyltransferase	Chr10	41007940	41009451
Glyma.20G213700	Isoflavone 7-O-methyltransferase	Chr20	45010959	45012474
Glyma.10G176600	Isoflavone 7-O-methyltransferase	Chr10	41013027	41014294
Glyma.18G269600	Isoflavone 7-O-methyltransferase	Chr18	55329239	55333846
Glyma.10G176700	Isoflavone 7-O-methyltransferase	Chr10	41027227	41028670
Glyma.20G027800	Isoflavone reductase homolog P3-related	Chr20	3179955	3183453
Glyma.08G247100	Isoflavone-7-O-beta-glucoside 6''-O-malonyltransferase / Flavone/flavonol 7-O-beta-D-glucoside malonyltransferase	Chr08	21397696	21399123
Glyma.18G268200	Isoflavone-7-O-beta-glucoside 6''-O-malonyltransferase / Flavone/flavonol 7-O-beta-D-glucoside malonyltransferase	Chr18	55220523	55222462
Glyma.19G030800	Isoflavone-7-O-beta-glucoside 6''-O-malonyltransferase / Flavone/flavonol 7-O-beta-D-glucoside malonyltransferase	Chr19	3799941	3801335
Glyma.19G030700	Isoflavone-7-O-beta-glucoside 6''-O-malonyltransferase / Flavone/flavonol 7-O-beta-D-glucoside malonyltransferase	Chr19	3794404	3796426
Glyma.18G268400	Isoflavone-7-O-beta-glucoside 6''-O-malonyltransferase / Flavone/flavonol 7-O-beta-D-glucoside malonyltransferase	Chr18	55227777	55229508
Glyma.18G268500	Isoflavone-7-O-beta-glucoside 6''-O-malonyltransferase / Flavone/flavonol 7-O-beta-D-glucoside malonyltransferase	Chr18	55235304	55237037
Glyma.18G258000	Isoflavone-7-O-beta-glucoside 6''-O-malonyltransferase / Flavone/flavonol 7-O-beta-D-glucoside malonyltransferase	Chr18	54359057	54361006
Glyma.18G268100	Isoflavone-7-O-beta-glucoside 6''-O-malonyltransferase / Flavone/flavonol 7-O-beta-D-glucoside malonyltransferase	Chr18	55215336	55216775
Glyma.08G247200	Isoflavone-7-O-beta-glucoside 6''-O-malonyltransferase / Flavone/flavonol 7-O-beta-D-glucoside malonyltransferase	Chr08	21403223	21408105
Glyma.13G054000	Isoflavone-7-O-beta-glucoside 6''-O-malonyltransferase / Flavone/flavonol 7-O-beta-D-glucoside malonyltransferase	Chr13	15126544	15128286
Glyma.18G267900	isoflavone-7-O-methyltransferase (7-IOMT)	Chr18	55199483	55200950
Glyma.16G219500	Methylecgonone reductase	Chr16	37677371	37680638
Glyma.09G074900	Naringenin-chalcone synthase / Flavonone synthase	Chr09	8120501	8121567

Glyma.08G110400	Naringenin-chalcone synthase / Flavonone synthase	Chr08	8478834	8480215
Glyma.03G181700	Phenylalanine ammonia-lyase	Chr03	39362677	39366612
Glyma.02G309300	Phenylalanine ammonia-lyase	Chr02	48287101	48289732
Glyma.10G058200	Phenylalanine ammonia-lyase	Chr10	5328963	5333501
Glyma.20G180800	Phenylalanine ammonia-lyase	Chr20	41891638	41898451
Glyma.03G181600	Phenylalanine ammonia-lyase	Chr03	39351927	39355288
Glyma.10G209800	Phenylalanine ammonia-lyase	Chr10	44173311	44179454
Glyma.19G182300	Phenylalanine ammonia-lyase	Chr19	44118211	44122192
Glyma.14G005700	Polyketide reductase (PKR)	Chr14	463009	464543
Glyma.07G202300	SF71 – Dirigent protein 1-related	Chr07	37166660	37169027
Glyma.20G114200	Trans-cinnamate 4-monooxygenase (CYP73A)	Chr20	35592130	35594408
Glyma.02G236500	Trans-cinnamate 4-monooxygenase (CYP73A)	Chr02	42416141	42420467
Glyma.14G205200	Trans-cinnamate 4-monooxygenase (CYP73A)	Chr14	47041931	47046048
Glyma.10G275600	Trans-cinnamate 4-monooxygenase / Cinnamic acid 4-monooxygenas	Chr10	49830805	49832699
Glyma.11G010500	Trans-feruloyl-CoA synthase / Trans-feruloyl-CoA	Chr11	737013	741142
Glyma.01G232400	Trans-feruloyl-CoA synthase / Trans-feruloyl-CoA synthetase	Chr01	55890929	55894888
Glyma.11G091600	Trans-feruloyl-CoA synthase / Trans-feruloyl-CoA synthetase	Chr11	6929293	6933159
Glyma.13G372000	Trans-feruloyl-CoA synthase / Trans-feruloyl-CoA synthetase	Chr13	45701238	45704364
Glyma.15G001700	Trans-feruloyl-CoA synthase / Trans-feruloyl-CoA synthetase	Chr15	190985	194451
Glyma.17G064400	Trans-feruloyl-CoA synthase / Trans-feruloyl-CoA synthetase	Chr17	4952729	4957405
Glyma.17G064500	Trans-feruloyl-CoA synthase / Trans-feruloyl-CoA synthetase	Chr17	4974218	4977314
Glyma.17G064600	Trans-feruloyl-CoA synthase / Trans-feruloyl-CoA synthetase	Chr17	4988012	4993853
